# Self-Assembled Vesicles of Emissive Surfactants in Water: Structural Characterization and Photophysical Insights

**DOI:** 10.1021/acs.jpcb.5c06034

**Published:** 2025-12-05

**Authors:** Kyosuke Arakawa, Natsuna Hosokawa, Yuichi Takasaki, Taku Ogura, Koji Tsuchiya, Kenichi Sakai, Hideki Sakai

**Affiliations:** † Department of Applied and Pure Chemistry, 26413 Tokyo University of Science, 2641 Yamasaki, Noda, Chiba 278-8510, Japan; ‡ Anton Paar Japan K. K., Riverside Sumida 1F, 1-19-9, Tsutsumi-dori, Sumida-ku, Tokyo 131-0034, Japan; § Research Institute for Science and Technology, Tokyo University of Science, 2641, Yamazaki, Noda, Chiba 278-8510, Japan

## Abstract

Two novel emissive amphiphilic molecules with intramolecular charge transfer character (HAPMP and HAPEOP) were synthesized, and their self-assembled particles were obtained in an aqueous solution. SAXS analysis and TEM observation showed that these self-assemblies exhibited vesicle-like structures. Analysis using the indirect Fourier transformation (IFT) method for their bilayer structures indicated that HAPMP was arranged in a vertical orientation relative to the bilayer plane, whereas HAPEOP adopted a tilted orientation. The maximum emission wavelengths in their vesicle states were red-shifted compared with those in the monomeric state. Time-resolved emission spectroscopy revealed prolonged emission lifetimes, suggesting that they formed an excimer in the bilayers.

## Graphical abstract

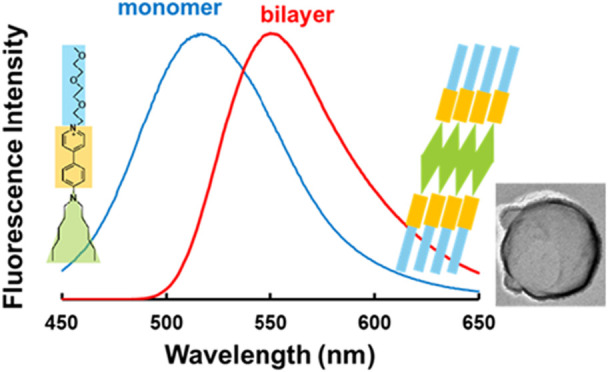

## 1 Introduction

Investigating techniques for controlling emissive colors of organic compounds is a continuing concern. Emission properties are strongly influenced not only by molecular structures but also by intermolecular orientations. Mechanochromism, in which the emission of organic crystals changes upon mechanical stimulus, exemplifies the close relationship between molecular packing and photophysical behavior.^1–4^ X-ray diffraction (XRD) of single crystals and powders provides powerful structural information that enables a correlation between molecular arrangement and emissive properties. Such precise analysis offered crucial insight into photophysical phenomena. However, these methods are inherently limited to crystalline solids, where thermal fluctuations in the molecular positions and configurations are minimal.

In contrast, molecular assemblies in aqueous media offer diverse supramolecular structures arising from complex intermolecular interactions. Hydrophobic interactions play a central role in the self-assembly of amphiphiles into micelles and vesicles, driven by the entropic stabilization of water molecules. Vesicles are particularly attractive because of their structural similarity to biomembranes and their potential applications as nanocarriers in drug delivery.^5–8^ In particular, emissive vesicles have drawn attention due to their ability to visualize localization and release encapsulated molecules, thus expanding their functional potential.^9^

To understand these soft matter systems, small-angle X-ray scattering (SAXS) provides a powerful tool for probing the structural features of colloids. Unlike XRD, SAXS does not provide atomic-level resolution, but it can reveal the size, shape, and overall organization of assemblies in solutions. The noninvasive nature of SAXS, requiring no drying or freezing, makes it particularly suitable for in situ characterization of delicate colloidal systems.^10–12^ Numerous studies of inorganic particles, proteins, and molecular assemblies have been reported. Nevertheless, despite reports on emissive vesicles,^13–15^ detailed structural evaluation from scattering patterns has remained limited.

In this study, we address this gap by applying SAXS in combination with indirect Fourier transformation (IFT), a model-free analysis that provides real-space information about the shape and size of colloids.^16–21^ Using this approach, we investigate the structural characteristics of novel emissive vesicles and discuss the correlation between their structures and their emissive behaviors. Two novel amphiphilic molecules were synthesized by introducing alkyl and poly­(oxyethylene) groups to 4-[4-(dimethylamino)­phenyl]-1-methylpyridinium (MAPMP)^22–24^ as the base molecular skeleton. MAPMP is not only synthetically accessible but also easily modified with hydrophilic and hydrophobic chains, making it a versatile platform for constructing functional self-assemblies.

## 2 Experimental Details

### 2.1 Materials

The chemicals for synthesis and identification of HAPMP and HAPEOP are noted down in the Synthesis Section of the Supporting Information (Figures S1 and S2). High-purity water was obtained using a Barnstead NANO pure Diamond UV system.

### 2.2 Surface Tension Measurements

Static surface tension measurements were performed using a Krüss K100C Wilhelmy auto surface tensiometer and a platinum plate at 25 °C. Surface tension measurements continued until the change in surface tension decreased to less than 0.01 mN m^–1^ per 90 s.

### 2.3 Absorption and Emission Spectra Measurements

Absorption spectra were measured by using a JASCO V-670 spectrophotometer. Emission spectra were measured by using a HITACHI F-2700 fluorescence spectrophotometer. For absorption and emission measurements, the HAPMP and HAPEOP solutions were placed in a quartz cell (the optical path length was 1.0 or 0.1 cm). The optical window with a width of 1.0 cm of the quartz cell, which has a 0.1 cm optical path length, was positioned at an angle of 45° to the excitation light source and detector.

### 2.4 Emission Quantum Yields

The value of quantum yields was measured on a Hamamatsu Photonics C13534–01 UV–NIR absolute PL quantum yield spectrometer.

### 2.5 Dynamic Light Scattering (DLS)

Particle size distributions were obtained by using an Anton Paar Litesizer 500 system for a 10 mM solution at 25 °C. HAPMP and HAPEOP solutions were placed in a plastic cell (1.0 cm × 1.0 cm).

### 2.6 Optical Microscopy Images

Optical microscopy images were obtained using an IX73 microscope (Olympus Optical Co., Ltd.). Polarized optical micrographs were obtained by using crossed polarizers. The resulting images were transferred to a computer equipped with a Moticam 2000 digital camera fitted with an eyepiece.

### 2.7 Freeze-Fracture Transmission Electron Microscopy (FF-TEM)

The morphologies of the molecular assemblies were observed using FF-TEM. A drop of the sample was rapidly frozen in liquid propane (<170 °C) using an EM CPC cryopreparation system (Leica Microsystems, Germany). The frozen sample was then fractured with a glass knife at −120 °C under vacuum (∼10^–5^ Pa) using a freeze-replica preparation apparatus FR-7000A (Hitachi High-Technologies Co., Ltd., Japan). A replica film of the fractured surface was formed by evaporating platinum/carbon at an angle of 45°, followed by carbon at an angle of 90° by using the apparatus. The replica film was removed from the apparatus and washed with chloroform/methanol (v/v = 2:1), acetone, and water. Subsequent to the placement of the replica film on a 400-mesh TEM copper grid, it was examined under a transmission electron microscope H-7650 (Hitachi High-Technologies Co., Ltd., Japan) at an accelerating voltage of 100 kV.

### 2.8 Time-Resolved Emission Spectrum Measurement

Emission lifetimes were measured using a C4780 ps fluorescence lifetime measurement system (Hamamatsu Photonics). A Nd^3+^ YAG laser (EKSPLA PL2210JE + PG-432, 25 ps FWHM, 1 kHz) was used for excitation. The excitation wavelength was 430 nm. Emission lifetimes were calculated by deconvolution of the excitation pulses in each measurement range. Lifetime calculations were performed with tail fit methods because the wavelength region of the emission spectrum is shifted sufficiently long compared with the excitation wavelength to be unaffected by the scattering blank of the laser pulse.

### 2.9 Quantum Chemical Calculations

Geometry optimization and molecular orbital calculations were performed using the density functional theory (DFT) method using the cam-B3LYP function and the 6-311+G­(d,p) basis set in a polar environment with water as the solvent. DFT calculations were conducted using GAUSSIAN 09. Visualization of each molecular orbital was performed with Jmol. The calculated optimal molecular structure of HAPMP and HAPEOP and their highest occupied molecular orbital (HOMO) and lowest unoccupied molecular orbital (LUMO) are shown in the Supporting Information (Figures S3 and S4).

### 2.10 Small-Angle X-ray Scattering (SAXS) Measurements

SAXS measurements were carried out at beamline BL08W in NanoTerasu. The photon energy of the incident beam was 8.0 keV. The X-ray wavelength was 1.54 Å. The acquisition time was constant at 1 min for all samples. The sample solutions were enclosed in borosilicate glass capillaries (AS ONE, Japan; Φ1.5 mm, 0.01 mm thickness) and measured at 25 °C. The scattered intensity was collected using two-dimensional (2D) scattering patterns from an EIGER2 1 M series detector (Dectris, Switzerland) at a sample to detector distance of 800 mm. The q-range of this setup was 0.033 ≤ *q* ≤ 2.7 nm^–1^. All X-ray scattering curves were corrected for background scattering from the capillary and water.

### 2.11 SAXS Pattern Analysis

Dimensional reduction of row data and background subtraction were carried out with SAXS analysis (Anton Paar Co.). The structure analysis of bilayers in the obtained vesicle-like particles was performed using the indirect Fourier transformation (IFT) method.^16–21^ The X-ray scattering intensity obtained from SAXS analysis is determined using the following equation

1 I(q)=nP(q)S(q)

where *n* is the number density of particles, *P*(*q*) is the average form factor, and *S*(*q*) is the structure factor. *P*(*q*) provides information related to the internal structure, size, and shape of the particles, while *S*(*q*) represents the interactions among the particles. When the measured particle is a vesicle, an interlamellar interaction in the vesicles corresponds to *S*(*q*). Because an inherent pattern as a structure factor was not observed in the obtained SAXS patterns, it is assumed that the obtained vesicles are unilamellar vesicles or the number of stacked bilayer membranes was small. Therefore, *S*(*q*) was hypothesized to be 1. The value of *q* indicates the magnitude of the scattering vector and is given by

2 $$q=\frac{4\pi }{\lambda }\sin\theta /2$$

where λ and θ represent the wavelength and scattering angle of the scattered X-rays, respectively. When the particle is a flat structure such as a bilayer, the form factor *P*(*q*) is given by the cosine transformation of the thickness distribution function, *p*_*t*_ (*r*), as

3 $$P_{t}\left(q\right)=2\int_{0}^{\infty }p_{t}\left(r\right)\cos\left(\mathrm{qr}\right)dr$$

where *P*_*t*_(*q*) is the form factor of a bilayer, *p*_*t*_(*r*) is the pair distance distribution function (PDDF) of a bilayer, and the thickness of the bilayer, *D*_max_, can be estimated from the distance at which *p*_*t*_(*r*) converges to zero.

## 3 Results and Discussion

### 3.1 Interfacial Activity

Synthesized molecular structures and concentration-dependent interfacial tensions at the air–water interface of solutions containing HAPMP and HAPEOP are shown in Figure 1(a–d), respectively. The interfacial tensions of both solutions decreased on increasing the concentration under diluted conditions and reached constant values above 1.7 mmol/L for HAPMP and 0.5 mmol/L for HAPEOP. This concentration dependence indicates that both molecules exhibit surfactant-like activity; in other words, these molecules have hydrophilic and hydrophobic parts. Figure 1e,f shows increases in turbidity and impairment of transparency above the critical aggregation concentrations (CACs). The observed turbidity suggests that some aggregates existed in the aqueous solutions; these aggregates are not micelles but larger supramolecular assemblies with a size of several hundred nm. The lower CAC of HAPEOP than HAPMP implies that HAPEOP has lower water solubility due to the substitution of a poly­(oxyethylene) group.

**Figure 1:**
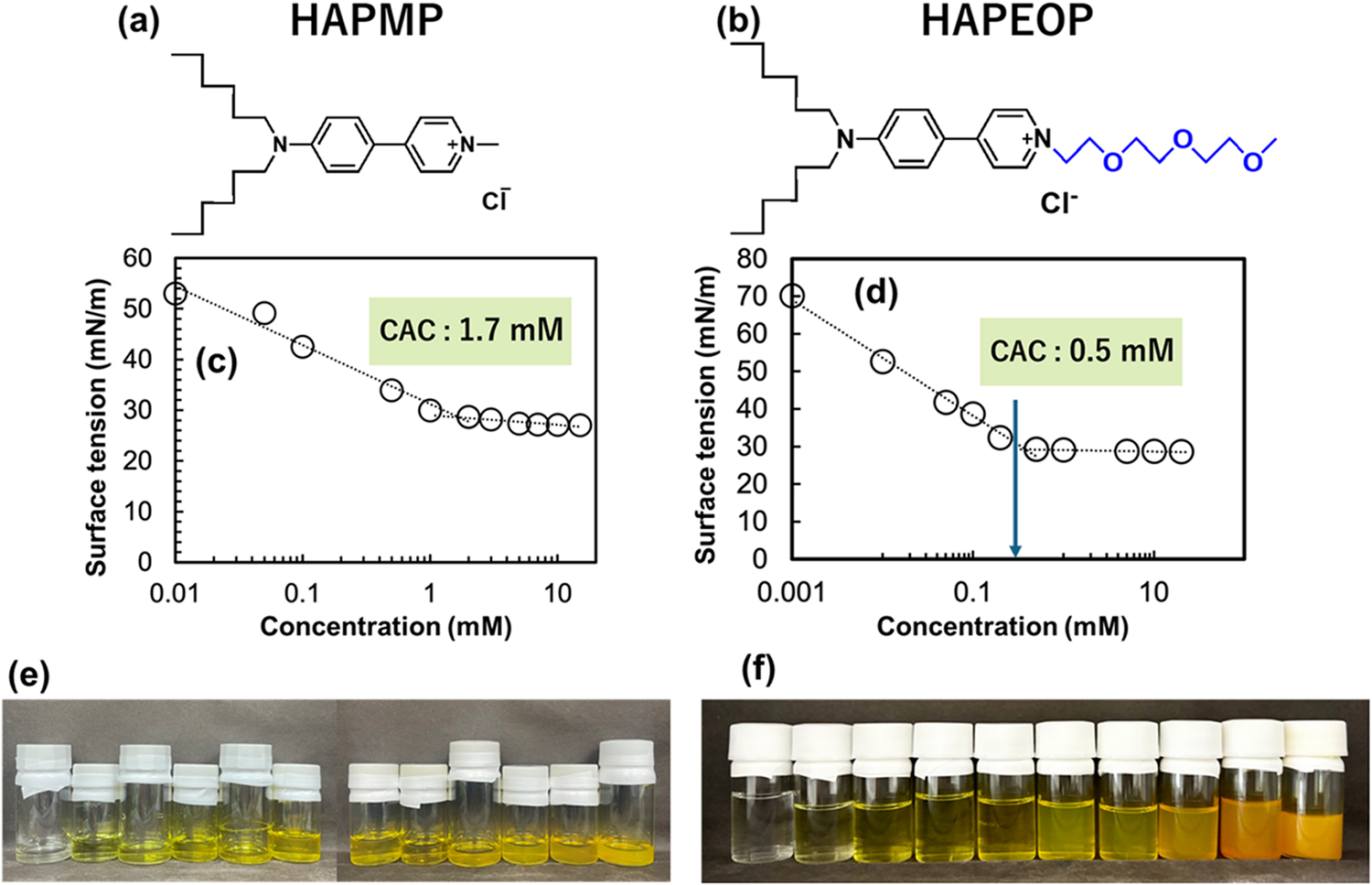
(a, b) Molecular structures of HAPMP and HAPEOP. (c, d) Concentration dependency of surface tensions for solutions containing HAPMP and HAPEOP at 0.001, 0.01, 0.05, 0.1, 0.2, 0.5, 1, 5, 10, and 20 mmol/L. (e, f) Solutions containing HAPMP and HAPEOP at 0.001, 0.01, 0.05, 0.1, 0.2, 0.5, 1, 5, 10, and 20 mmol/L.

### 3.2 Morphology and Size of Self-Assemblies

The size distributions of both dispersions at 10 mmol/L HAPMP and HAPEOP with dynamic light scattering (DLS) measurement are shown in Figure 2a,b. The hydrodynamic diameters are distributed around several hundred nanometers, and both average diameters were around 200 nm. Freeze-fracture transmission electron microscopy (FF-TEM) images also show some similar sizes of particles. These results show that the self-assembled structures are not micelles, which are generally only a few nanometers in size. While Figure 2c shows several distorted spherical particles for HAPMP, Figure 2d shows spherical particles with smooth outlines. Figure 2e,f shows the bright-field images and polarized light images for both dispersions. Spherical particles with sizes of several micrometers were observed in both images. It is assumed that very few percentages of the particles with enough sizes for optical microscopy observation were detected. Maltese crosses were found in both polarized light images, suggesting that observed particles have a structure like multilamellar vesicles. HAPMP and HAPEOP are composed of one pyridinium ring and one phenyl ring and two alkyl groups. T. Kunitake and co-workers investigated the self-assemblies of surfactants with double long alkyl chains.^25^ In many cases, surfactants with double long alkyl chains formed vesicle-like assemblies. Bacause HAPMP and HAPEOP also have the double-alkyl chains, it is reasonable to form a vesicle.

**Figure 2:**
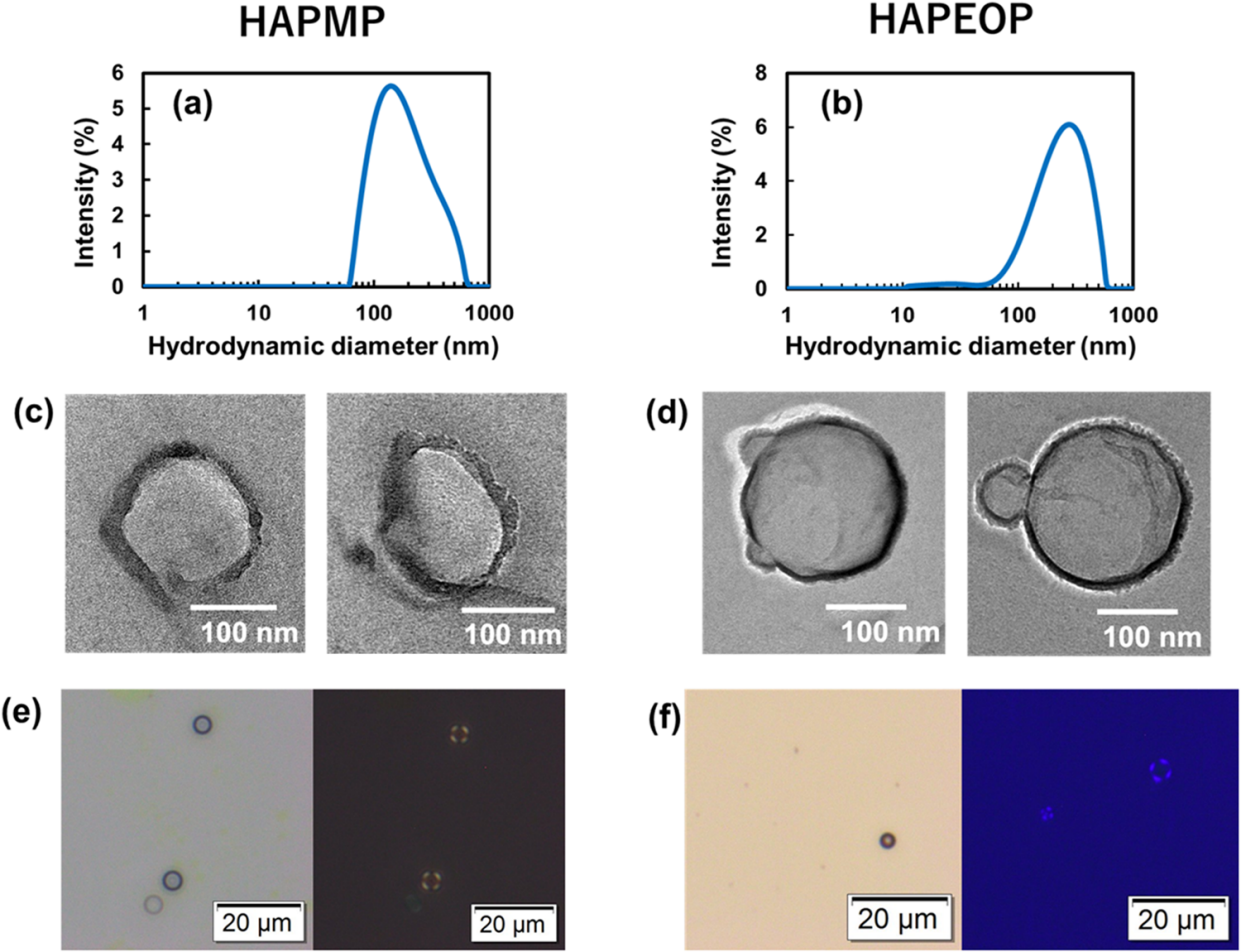
(a, b) Size distribution of the formed particles in HAPMP and HAPEOP solutions at 10 mmol/L with DLS measurements. (c, d) FF-TEM images of the particles. (e, f) Bright-field images and polarized light images for both dispersions.

### 3.3 SAXS Analysis for Thickness Evaluation of the Bilayers

SAXS patterns of HAPMP and HAPEOP dispersions are shown in Figure 3a,b. Broad peaks were confirmed at around 0.2 nm^–1^ in both patterns. A repeated pattern that is considered a structure factor was not found. Both scattering patterns almost follow the dotted line (*I­(q)* = *C* *q* ^–2^), where *C* is the arbitrary constant, which indicates that the scatters have a plate-shaped structure,^26^ such as a bilayer. This suggests that the particles have a plate-shaped structure. It should be noted that this means the particles have a plate-shaped structure in the particle, not that their overall shapes are platelike, considering the measured scattering range is around 1 nm^–1^. To summarize the results of DLS, TEM, and SAXS, the particles have a bilayer structure with single nm thickness, and the shape is like a sphere with around 200 nm diameter. It shows that the self-assemblies of HAPMP and HAPEOP are a vesicle-like structure.

**Figure 3:**
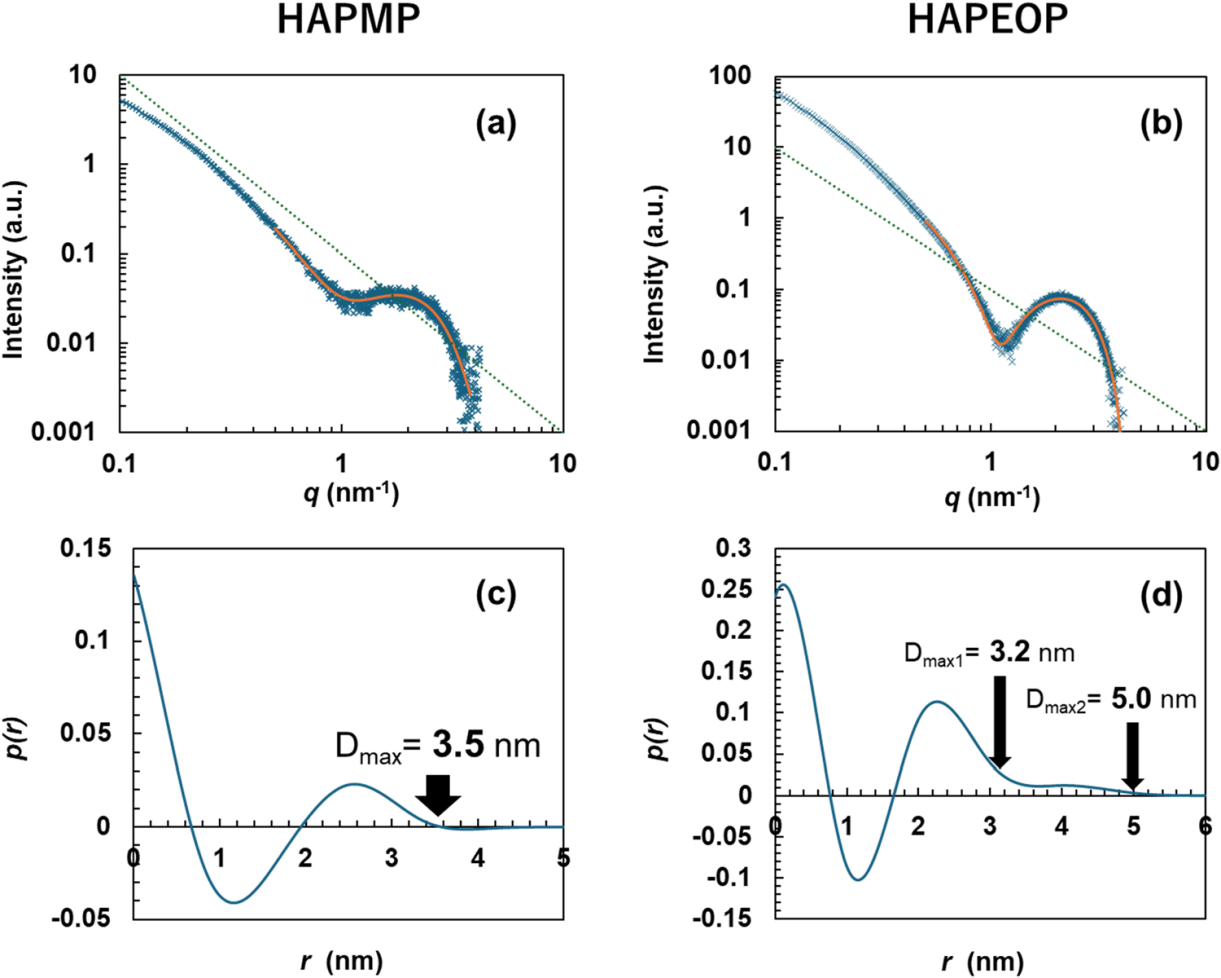
(a, b) SAXS patterns for the HAPMP and HAPEOP dispersions: measured scattering data (blue cross); fitting curve reproduced by the calculated PDDF curve (orange line); and *I­(q)* = *C* *q* ^–2^ (dotted blue line). (c, d) Thickness pair distance distribution functions.

Structure analysis with the IFT method was carried out, and the thickness pair distance distribution functions (PDDFs, *p­(q)*) are shown in Figure 3c,d. The resulting fit functions obtained by the IFT method for HAPMP and HAPEOP are shown in Figure 3a,b as orange solid lines. The fitting curves showed good agreement with the experimental data in the range of 0.5 to 4 nm^–1^. The bilayer thickness (*D*_max_) was determined from the distance at which *p­(r)* of the PDDF curve converged to zero. In the case of the bilayer composed of HAPMP, the thickness is around 3.5 nm. In the case of that of HAPEOP, the PDDF function has a similar trend with that of HAPMP up to around 3.2 nm and then the curve with weak intensity reached *p­(r)* = 0 around 5 nm. These features were interpreted as arising from two distinct thicknesses within the bilayer. The former corresponds to a thickness of approximately 3.2 nm (*D*_max1_), attributed to the portion of the bilayer composed of the two aromatic rings and two hexyl chains in HAPEOP. The latter reflects a total thickness of 5.0 nm (*D*_max2_), suggesting that the poly­(oxyethylene) group of HAPEOP extends outside of the bilayer. The small amplitude up to 5 nm may reflect a small distinct of electron density fluctuation between the bulk water and the outside layer where poly­(oxyethylene) was located. It is assumed that the smaller thickness (3.2 nm) than that of the HAPMP bilayer (3.5 nm) indicates that HAPEOP was tilted to the bilayer plane.

### 3.4 UV–Vis Absorption and Emission Behavior

UV–vis absorption spectra of HAPMP and HAPEOP under diluted conditions and in the aggregated states are shown in Figure 4a,b. Maximum absorption wavelengths (λ_max_) were blue-shifted about 10 nm upon the self-assemblies. The solvation effect for the absorption spectra of MAPMP, which is the core structure of HAPMP and HAPEOP, was investigated and as the polarity of the solvent decreases, the maximum absorption shifts to a longer wavelength.^24^ Do the blue shifts by forming the vesicles suggest that the environment surrounding the molecule in the bilayer structure is like that of high-polarity solvents? Both molecules in the vesicles are surrounded by themselves, and their electronic dipole moments arrange in the same direction in the ground state. This situation is not likely to stabilize their frontier orbitals, as high-polarity solvents stabilize the orbitals by solvation. Therefore, it is unlikely that the observed blue shift caused by aggregation arises from the solvation effect. Thus, the blue shift suggested that the formation of H-aggregation was based on Kasha’s theory.^27^

**Figure 4:**
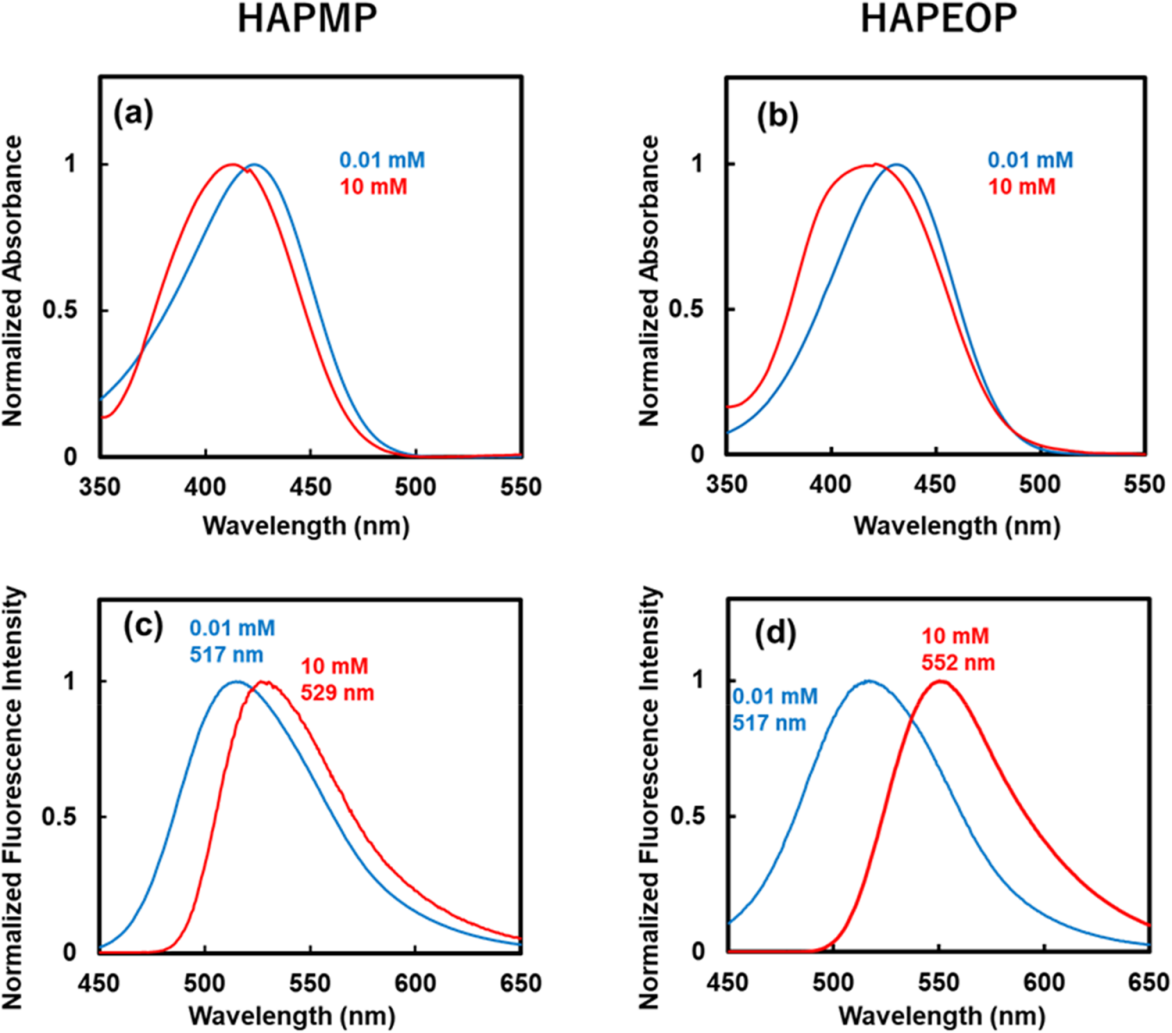
Absorption and emission spectra of (a, c) HAPMP and (b, d) HAPEOP in monomeric (0.01 mM) and self-assembled states (10 mM) in water. The excitation wavelength was 430 nm.

Emission spectra of HAPMP and HAPEOP under diluted conditions and in the aggregated state are shown in Figure 4c,d. Maximum emission wavelengths were red-shifted approximately 12 nm for HAPMP and 35 nm for HAPEOP upon the self-assemblies. The emission quantum yields of HAPMP are 0.003 and 0.003 at 0.01 and 10 mmol/L, respectively; those of HAPEOP are 0.006 and 0.015 at 0.01 and 10 mmol/L, respectively. Considering general H-aggregates are nonemissive or weak-emissive states because of the forbidden excited states and the Stokes shift generally tends to be small, it is not likely that the red-shifted emissions are attributed to the H-aggregations.

The time-resolved emission intensity for the aqueous solution containing HAPMP or HAPEOP in monomeric (0.01 mM) and vesicle states (10 mM) are shown in Figures 5a,b and 6a,b. Emission lifetimes of HAPMP and HAPEOP in the monomeric state were very short and almost comparable. It is considered that the short lifetime arose from the charge transfer property. On the other hand, these molecules in their self-assembled vesicles exhibited significantly prolonged emission lifetimes. The time-resolved emission spectra were integrated over two separate time windows: an early window immediately following excitation and a delayed window starting after a short time interval (Figures 5c and 6c). Because the integrated spectra of the short-lived components were similar to the steady-state emission band under monomer conditions shown in Figure 4c,d, it is considered that the emissive mechanism of the short-lived components is similar to that of the monomers. And the λ_max_ of the integrated spectra of the long-lived components were also almost similar to that of the steady-state emission band under the vesicle-forming conditions shown in Figure 4c,d. The blue-shifted absorption band, the red-shifted emission band, and the elongation of the emission lifetime that are accompanied by molecular aggregation have been reported in several studies.^28,29^ Such emission behaviors were assumed by excimer formation.^28,29^ In this work, the spatial distributions of HOMOs and LUMOs of neighboring molecules in the bilayers were close to each other, suggesting the likelihood of excimer formation.

**Figure 5:**
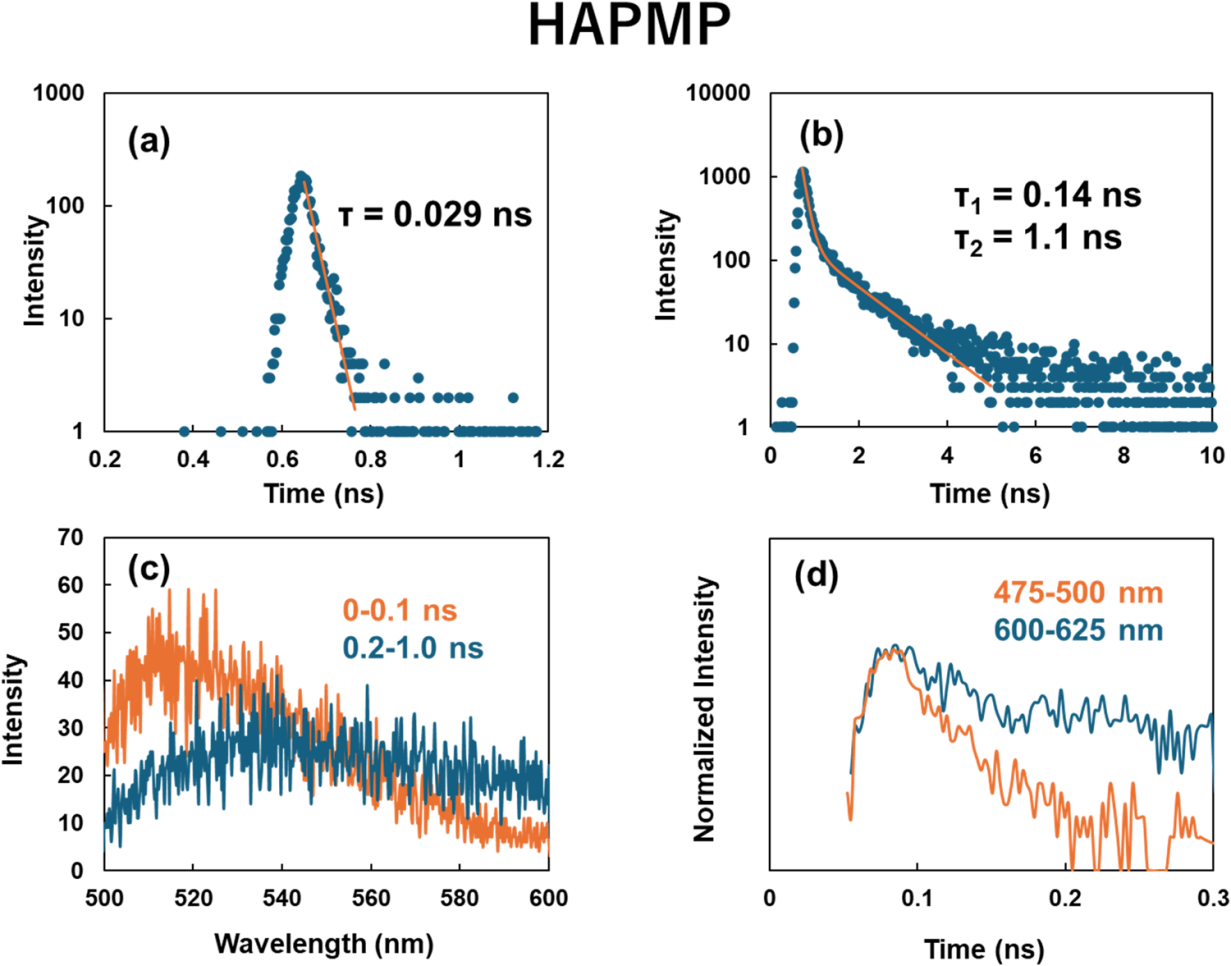
Time-resolved emission intensity for the HAPMP aqueous solution at (a) 0.01 and (b) 10 mM. (c) Integrated emission spectrum at (orange) the period immediately after excitation and (blue) after a short delay following excitation for the same sample as in panel (b). (d) Time-resolved emission intensity integrated at (orange) 475–500 nm and (blue) 600–625 nm for the same sample as in panel (b). The excitation wavelength was 430 nm.

**Figure 6:**
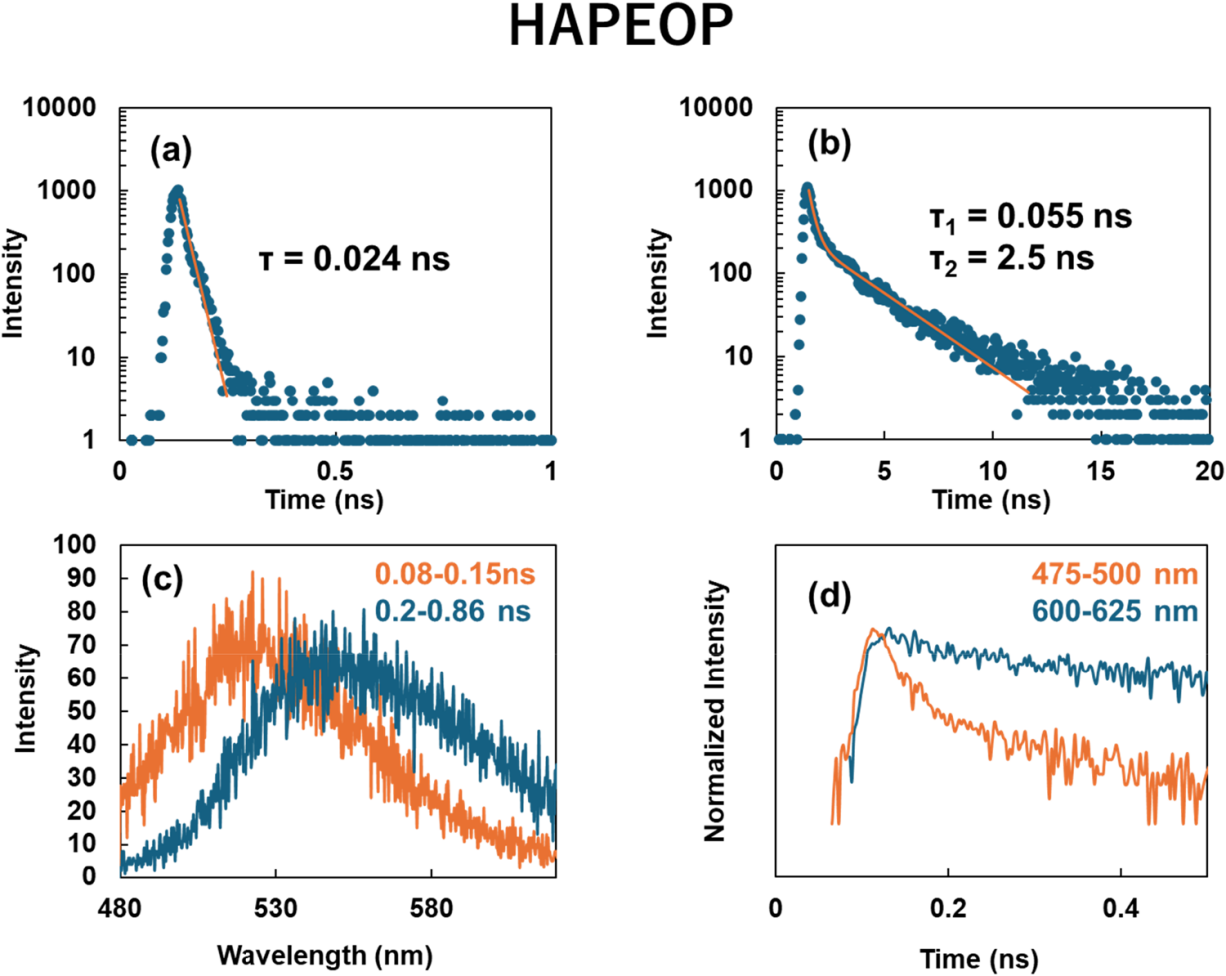
Time-resolved emission intensity for the HAPEOP aqueous solution at (a) 0.01 and (b) 10 mM. (c) Integrated emission spectrum at (orange) the period immediately after excitation and (blue) after a short delay following excitation for the same sample as in Figure 5b. (d) Time-resolved emission intensity integrated at (orange) 475–500 nm and (blue) 600–625 nm for the same sample as in Figure 5b. The excitation wavelength was 430 nm.

Time dependences of the emission intensity integrated over the ranges of 475–500 and 600–625 nm are shown in Figures 5d and 6d for HAPMP and HAPEOP, respectively, to evaluate the rise delay typically observed in general excimer emissions. However, no significant delay of the long-wavelength component relative to the short-wavelength component was observed. It is assumed that the molecular aggregation suppressed the degree of freedom of the rotation and vibration of excited molecules, and the sufficient close distance between neighboring molecules shortens the required time for the formation of the excimer from the Franck–Condon state. Thus, it is impossible to observe the process of excimer formation with the time resolution of the instruments used in this work.

### 3.5 Estimation of the Detailed Self-Assembled Structure

In this section, we aim to understand the detailed structure of the self-assembled bilayers in order to clarify why the bilayer formation causes the larger red shift in the emission band and the greater elongation of the emission lifetime of HAPEOP than that of HAPMP. Considering that the calculated thickness of the bilayer composed of HAPMP is around 3.5 nm and the molecular length of HAPMP is around 1.7 nm, a vertical molecular orientation to the bilayer plane is suggested (Figure 7a). In the case of HAPEOP, the thickness which eliminated the contribution of poly­(oxyethylene) groups is about 3.2 nm, and considering the single molecular length is 1.7 nm, which also ignores poly­(oxyethylene) parts, the averaged orientation angle was estimated to be about 70° (Figure 4b). It is assumed that the steric repulsion among the poly­(oxyethylene) groups causes the tilted orientation of HAPEOP. It is well known that alterations of absorption bands due to the formation of H- or J-aggregation are related with the orientation angles between the neighboring molecules (eq 2).^27,30,31^

4 $$\Delta E=\frac{\mu^{2}\left(1-3\cos^{2}\theta \right)}{4\pi \varepsilon \varepsilon_{0}R^{3}}$$

5 $$R^{\prime }=R\sin\theta$$

**Figure 7:**
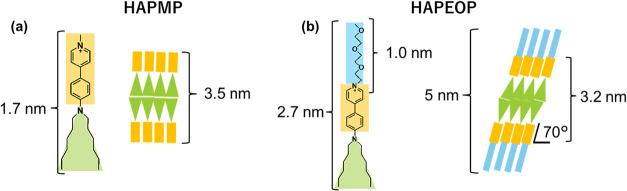
(a, b) Molecular lengths of HAPMP and HAPEOP and the estimated bilayer structure.

Δ*E* is the energy difference between the peak tops of the absorption spectra of the monomeric and aggregation states, μ is the dipole moment of the *S*_0_–*S*_1_ transition of molecules, θ is the orientation angle, ε and ε_0_ are the dielectric constants of vacuum and the medium, and *R* is the distance between the centers of the transition dipole moments of neighboring molecules. In order to discuss just an energy shift caused by the formation of the H-aggregate, minimization of the contribution of the solvation effect is required. We hypothesized that the solvation effect that HAPMP and HAPEOP felt in the bilayers is similar to that in toluene solution because the segments in HAPMP and HAPEOP which are related to the absorption behavior were surrounded by the aromatic parts of themselves. Δ*E* values of both molecules were calculated by using the energy difference between the λ_max_ of absorption spectra in toluene and in the bilayers. The absorption spectra in toluene of HAPMP and HAPEOP are shown in Figures S5 and S6. λ_max_ values of HAPMP and HAPEOP in toluene were 424 and 426 nm. λ_max_ values of HAPMP and HAPEOP in the bilayers were 413 and 418 nm. μ for HAPMP was calculated by an experimental procedure in a previous report as 7.7 debye^32^ and ε was hypothesized as 2.5,^33^ which is the value for toluene. Based on these estimated values and eq 2, the values of R were calculated as 5.7 and 5.5 Å for HAPMP and HAPEOP, respectively.

In the previous section, it is assumed that the emission behavior change induced by the bilayer assembly was due to excimer formation. An excimer is a short-lived aggregate with sharing of electrons between ground-state and excited-state molecules. Therefore, the closer proximity of the frontier orbitals leads to the formation of more stabilized bonding and more destabilized antibonding orbitals, resulting in a larger red shift and elongation lifetime in the emission. Thus, here, the vertical distance between the neighboring molecules is important. Figure 8 shows the vertical distance between the planes of their aromatic segments; here, *R*′ and the distance between neighboring transition dipole moments *R* are related with eq 3. *R*′ values were calculated as 5.7 and 5.2 Å for HAPMP and HAPEOP, respectively. In the case of HAPMP, *R* = *R*′. It is assumed that *R*′ of HAPEOP in the vesicle is shorter than that of HAPMP because the tilted arrangement of HAPEOP mitigates the repulsion between pyridinium cations. Therefore, the bonding and antibonding molecular orbitals of the HAPEOP excimer are more stabilized and destabilized than those of HAPMP.

**Figure 8:**
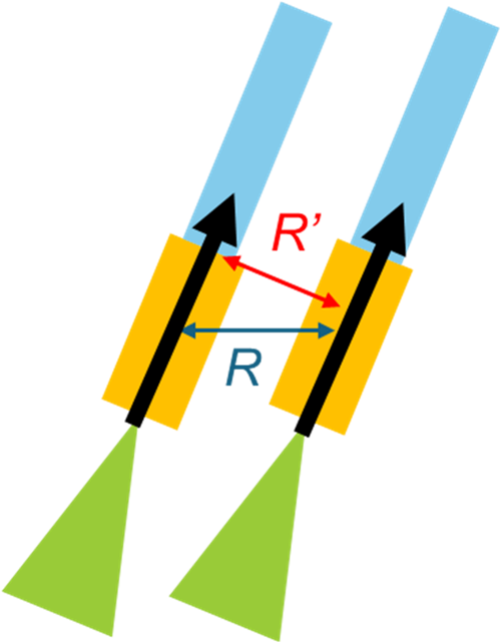
Center-to-center distance (*R*; blue double-headed arrow) and vertical distance (*R*′; blue double-headed arrow) between neighboring molecules. Black arrows indicate the transition dipole moment.

While this estimation is effective to qualify the comparison between the two bilayer structures, the calculated absolute values must be interpreted with caution. Actually, considering the general molecular distance in organic crystals, the estimated *R*′ values are too large. In order to obtain a highly accurate value, it is necessary to use an appropriate dielectric constant and to remove the solvent effect on Δ*E* completely. Furthermore, it should be noted that in contrast to crystals, the bilayer structures exhibit thermal fluctuations, and the estimated bilayer thickness and orientation angle only represent the averaged values.

## 4 Conclusion

Investigation of the structure and emissive behavior of the newly prepared vesicle-like particles was carried out. SAXS analysis with the IFT method indicated that HAPMP was vertically arranged and HAPEOP was arranged in a tilted orientation to their bilayer plane. A rough estimation of the molecular distance in the aggregates based on the energy shifts caused by H-aggregation formation assumed that the distance of HAPEOPs was closer than that of HAPMPs. The red shift of λ_max_ in the emission spectra caused by the vesicle formation and the emissive lifetime elongation indicated excimer formation in the vesicles. It is speculated that the larger Stokes shift of HAPEOP in the vesicle than that of HAPMP is driven by the closer molecular distance of HAPEOP. In the present study, we tried to approach detailed information on the self-assemblies including a constant degree of thermal fluctuation by the combination of SAXS pattern analysis and spectrophotometry. Obtaining appropriate and definitive information, which can be approached in the case of organic crystals, was not realistic; however, it seems to be a helpful approach for qualitative understanding.

## Supplementary Material



## Notes

Details of synthesis and characterization for HAPMP and HAPEOP; ^1^H NMR spectrum of HAPMP in CDCl_3_ at room temperature (Figure S1); ^1^H NMR spectrum of HAPEOP in CDCl_3_ at room temperature (Figure S2); optimal structure of HAPMP at the ground state and the HOMO and LUMO (Figure S3); optimal structure of HAPEOP at the ground state and the HOMO and LUMO (Figure S4); absorption spectrum of HAPMP in toluene (Figure S5); and absorption spectrum of HAPEOP in toluene (Figure S6) (PDF)
