# Supplementary material for: Self-Assembled Vesicles of Emissive Surfactants in Water: Structural Characterization and Photophysical Insights
Source: J Phys Chem B. 2025 Dec 5;129(50):12988–96. doi: 10.1021/acs.jpcb.5c06034 (PMC12720226; doi:10.1021/acs.jpcb.5c06034)
Supplement: Supplementary file 1 [file jp5c06034_si_001.pdf]

Supporting Information

Self-Assembled Vesicles of Emissive Surfactants in Water: Structural Characterization and Photophysical Insights

Kyosuke Arakawa[1*], Natsuna Hosokawa[1], Yuichi Takasaki[2], Taku Ogura[3], Koji Tsuchiya[3], Kenichi Sakai[1,3], Hideki Sakai[1,3*]

[1] Department of Applied and Pure Chemistry, Tokyo University of Science, 2641 Yamasaki, Noda-city, Chiba, 278-8510, Japan

2 Anton Paar Japan K. K., Riverside Sumida 1F, 1-19-9, Tsutsumi-dori, Sumida-ku,

Tokyo 131-0034, Japan

[2] Research Institute for Science and Technology, Tokyo University of Science, 2641, Yamazaki, Noda, Chiba 278-8510, Japan

* Corresponding author

E-mail: k-arakawa@rs.tus.ac.jp

Synthesis

HAPMP was synthesized by the following scheme.

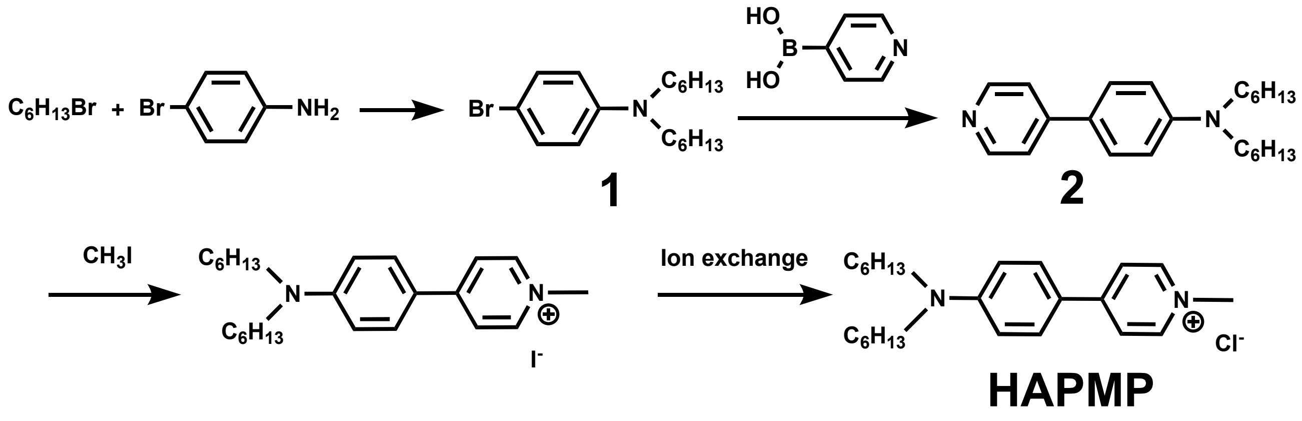

Scheme S1. Synthesis scheme.

4-Bromo-*N,N*-diehexylbenzenamine (1)

N 1-bromohexane (TCI Co.) (11.5 g, 66 mmol), 4-Bromoaniline (TCI Co.) (5.2 g, 30 mmol), Tetraethylammonium Iodide (TCI Co.) (1.1 g, 3.0 mmol), and 8 N NaOH(aq) (Wako Co.) (30 mL) were stirring at 373 K for 48 hours. Cooled the reaction mixture to room temperature. Separated water and organic layer and collected organic layer. After removing the organic solvents, the crude product was purified by chromatography over silica gel (hexane). The yellow liquid was obtained (yield is 92 %). (^{1}H NMR in $CDCl_3$):δ=7.23 (d, 11.5 Hz, 2H), 6.48 (d, 11.5 Hz, 2H), 3.19 (t, 9.5 Hz, 4H), 1.53(s, 4H), 1.29 (m, 12H), 0.88 (s, 6H).

***N,N*-Dihexyl-4-(4-pyridinyl)benzenamine (2)**

4-Bromo-*N,N*-diehexylbenzenamine (1) (5.0 g, 16 mmol), Pyridin-4-ylboronic acid (2.2 g, 18 mmol), Tetrakis(triphenylphosphine)palladium(0) (0.85g, 0.7 mmol), K_2CO_3 (3.53 g, 26 mmol) were dissolved in methanol (100 mL), Tetrahydrofuran (100 mL), water (25 mL) under nitrogen gas atmosphere. Stirred the mixture for 24 hours at 393 K. Cooled the reaction mixture to room temperature. Separated water and organic layer and collected organic layer. After removing the organic solvents, the crude product was purified by chromatography over silica gel (hexane: ethyl acetate = 8:1). The yellow liquid was obtained (yield is 39 %). (^{1}H NMR in $CDCl_3$) : δ=8.53 (d, J=8.0 Hz, 2H), 7.54 (d, J=11.5 Hz, 2H), 7.44 (d, J=8.0 Hz,

2H), 6.69 (d, J=11.0Hz, 2H), 3.30 (t, J=9.3 Hz, 4H), 1.61 (s, 4H) , 1.27 (m, J=12.4 Hz, 12H) 0.88 (s, 6H).

4-[4-(dihexylamino)phenyl]-1-methylpyridinium Chloride (HAPMP)

N,*N*-Dihexyl-4-(4-pyridinyl)benzenamine (2) (1.2 g, 3.6 mmol) and iodomethane (TCI Co.) (2.0 mL) were dissolved in acetonitrile (30 mL). Stirred the mixture for 24 hours at 353 K. Cooled the reaction mixture to room temperature. After removing solvents and iodomethane, the counter anion was exchanged from I^- to Cl^- with an ion-exchange resin (Organo, Amberlite IRA-400J Cl), and the yellow liquid was obtained (yield is 40 %). (^{1}H NMR in $CDCl_3$) : δ=8.82 (d, *J*=9.0 Hz, 2H), 7.97 (d, J=9.0 Hz, 2H), 7.71 (d, J=11.5 Hz, 2H), 6.72 (d, J=11.5 Hz, 2H), 4.45 (s, 3H), 3.36 (t, J=9.8 Hz, 4H), 1.62 (s, 4H), 1.34 (d, J=5.0 Hz, 6H), 0.91 (t, J=8.5 Hz, 6H). MS(FAB), m/z (%): 353.5 (100) [$C_{23}H_{37}N_2$].

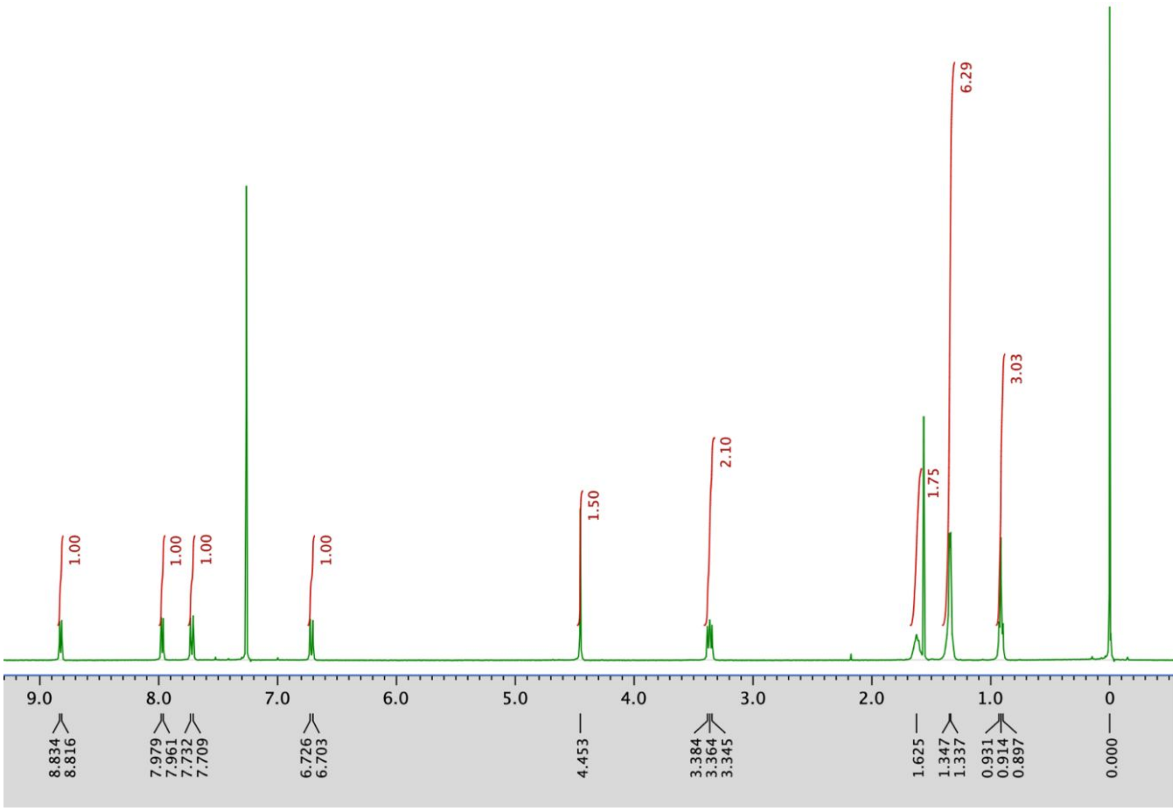

Figure S1. ^{1}H NMR spectrum of **HAPMP** in $CDCl_3$ at room temperature.

HAPEOP was synthesized by the following scheme.

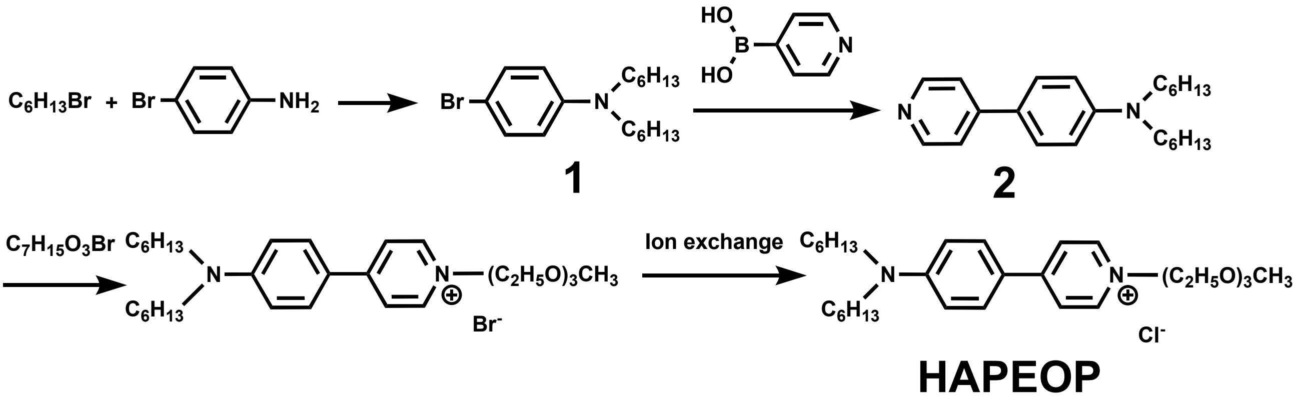

Scheme S2. Synthesis scheme.

4-Bromo-*N,N*-diehexylbenzenamine (1)

N 1-bromohexane (TCI Co.) (11.5 g, 66 mmol), 4-Bromoaniline (TCI Co.) (5.2 g, 30 mmol), Tetraethylammonium Iodide (TCI Co.) (1.1 g, 3.0 mmol), and 8 N NaOH(aq) (Wako Co.) (30 mL) were stirring at 373 K for 48 hours. Cooled the reaction mixture to room temperature. Separated water and organic layer and collected organic layer. After removing the organic solvents, the crude product was purified by chromatography over silica gel (hexane). The yellow liquid was obtained (yield is 92 %). (^{1}H NMR in $CDCl_3$):δ=7.23 (d, 11.5 Hz, 2H), 6.48 (d, 11.5 Hz, 2H), 3.19 (t, 9.5 Hz, 4H), 1.53(s, 4H), 1.29 (m, 12H), 0.88 (s, 6H).

***N,N*-Dihexyl-4-(4-pyridinyl)benzenamine (2)**

4-Bromo-*N,N*-diehexylbenzenamine (1) (5.0 g, 16 mmol), Pyridin-4-ylboronic acid (2.2 g, 18 mmol), Tetrakis(triphenylphosphine)palladium(0) (0.85g, 0.7 mmol), K_2CO_3 (3.53 g, 26 mmol) were dissolved in methanol (100 mL), Tetrahydrofuran (100 mL), water (25 mL) under nitrogen gas atmosphere. Stirred the mixture for 24 hours at 393 K. Cooled the reaction mixture to room temperature. Separated water and organic layer and collected organic layer. After removing the organic solvents, the crude product was purified by chromatography over silica gel (hexane: ethyl acetate = 8:1). The yellow liquid was obtained (yield is 39 %). (^{1}H NMR in $CDCl_3$) : δ=8.53 (d, J=8.0 Hz, 2H), 7.54 (d, J=11.5 Hz, 2H), 7.44 (d, J=8.0 Hz, 2H), 6.69 (d, J=11.0Hz, 2H), 3.30 (t, J=9.3 Hz, 4H), 1.61 (s, 4H) , 1.27 (m, J=12.4 Hz, 12H)

0.88 (s, 6H).

4-[(1*E*)-2-[4-(Dihexylamino)phenyl]ethenyl]-1-[2-[2-(2-methoxyethoxy)ethoxy]ethyl]pyridinium Chloride (HAPEOP)

N,*N*-Dihexyl-4-(4-pyridinyl)benzenamine (2) (0.3 g, 0.8 mmol) and 1-Bromo-2-[2-(2-methoxyethoxy)ethoxy]ethane (TCI Co.) (0.2 g, 0.9 mmol) were dissolved in acetonitrile (30 mL). Stirred the mixture for 24 hours at 353 K. Cooled the reaction mixture to room temperature. After removing the organic solvents, DHAPEOP was dissolved in a saturated aqueous solution of NaCl and changed the counter ion from I^- to Cl^- (yield is 88 %). (^{1}H NMR in $CDCl_3$) : δ=8.60 (d, *J*=9.0 Hz, 2H), 7.94 (d, J=9.0 Hz, 2H), 7.73 (d, J=11.5 Hz, 2H), 6.71 (d, J=11.5 Hz, 2H), 4.64 (s, 2H), 3.98 (s, 2H), 3.65 (s, 2H), 3.59 (s, 2H), 3.54 (s, 2H), 3.48 (s, 2H), 3.34 (s, 7H), 1.61 (s, 4H), 1.34 (s, 12H), 0.91 (t, J=8.5 Hz, 6H). MS(FAB), m/z (%): 485.5 (100) [$C_{30}H_{49}N_2O_3$].

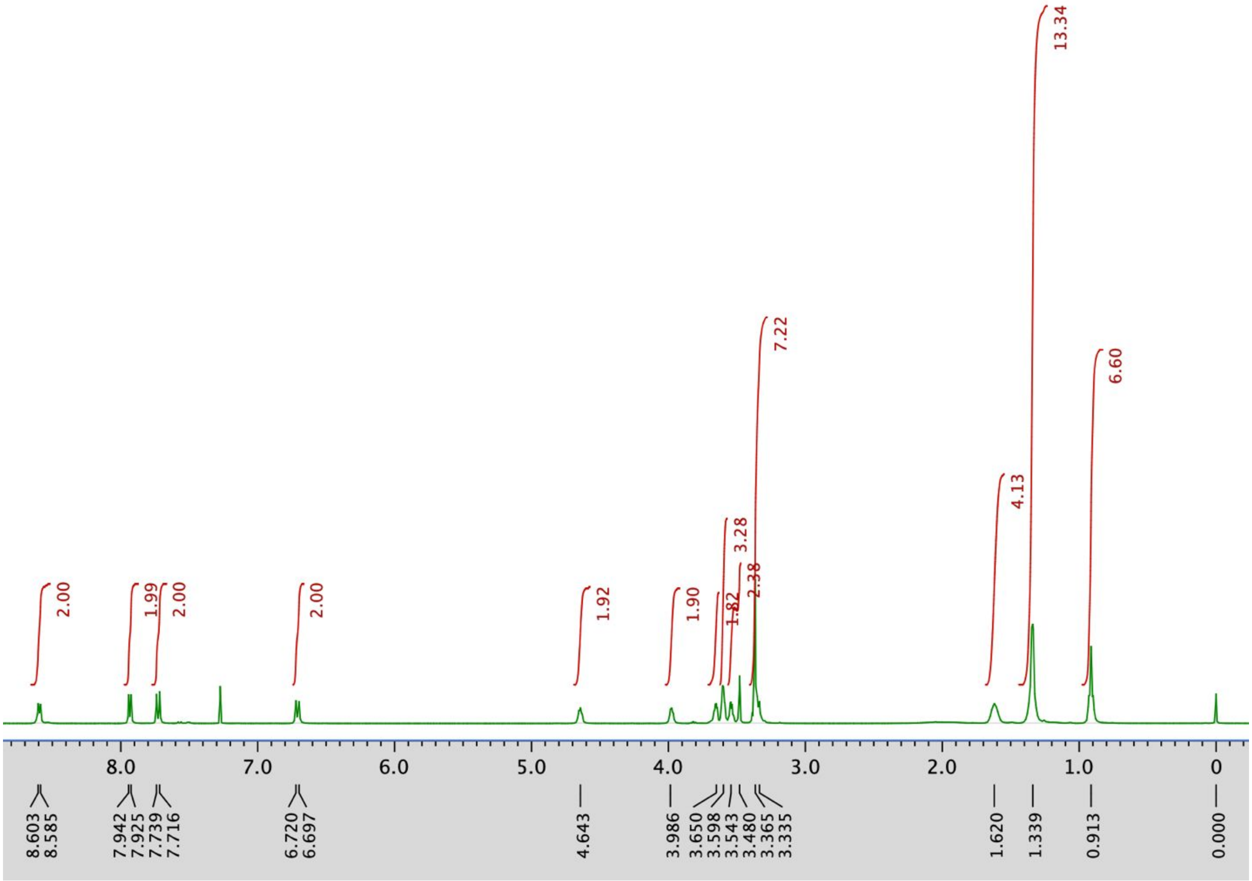

Figure S2. ^{1}H NMR spectrum of **HAPEOP** in $CDCl_3$ at room temperature.

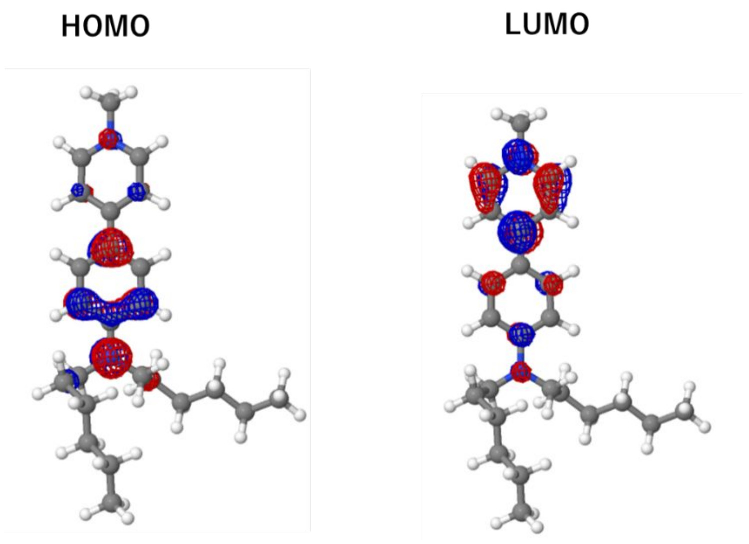

Figure S3. Optimal structure of **HAPMP** at ground state and HOMO and LUMO.

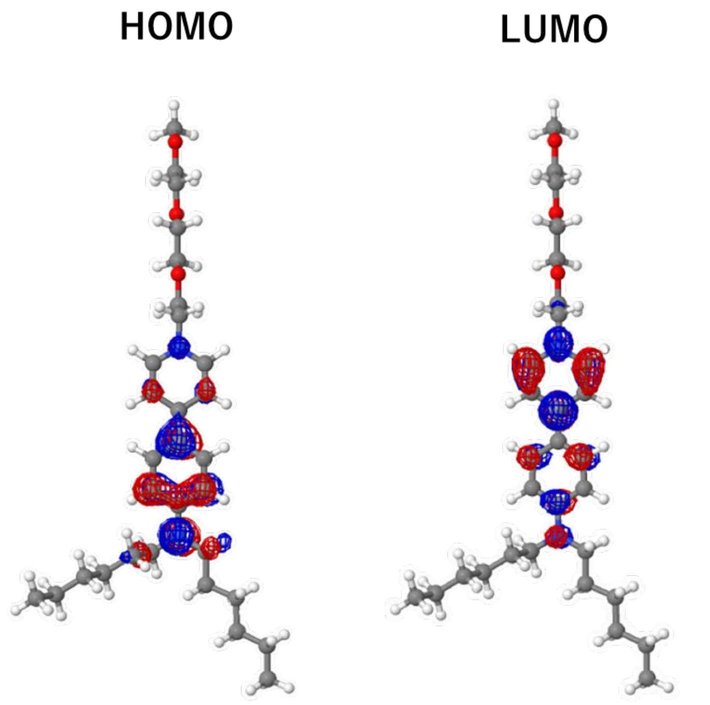

Figure S4. Optimal structure of **HAPEOP** at ground state and HOMO and LUMO.

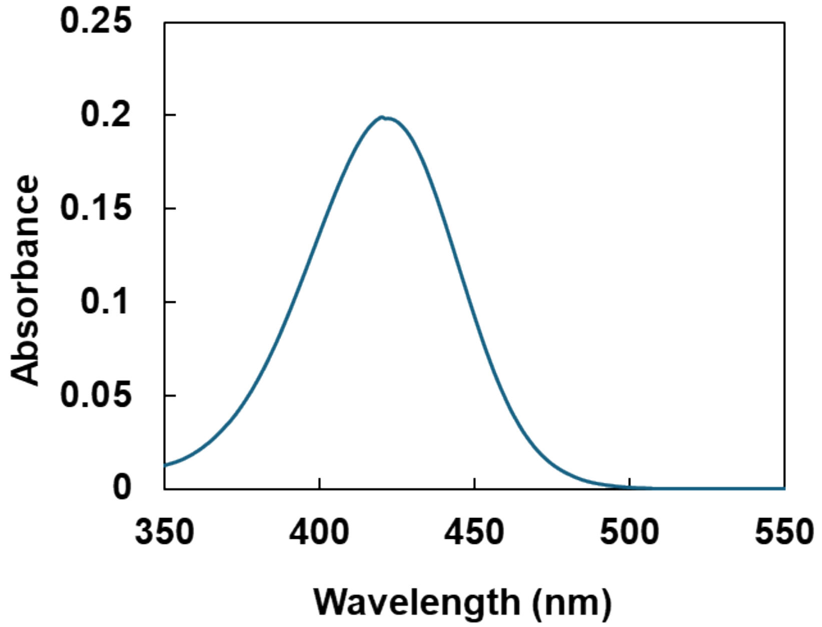

Figure S5. Absorption spectrum of HAPMP in toluene.

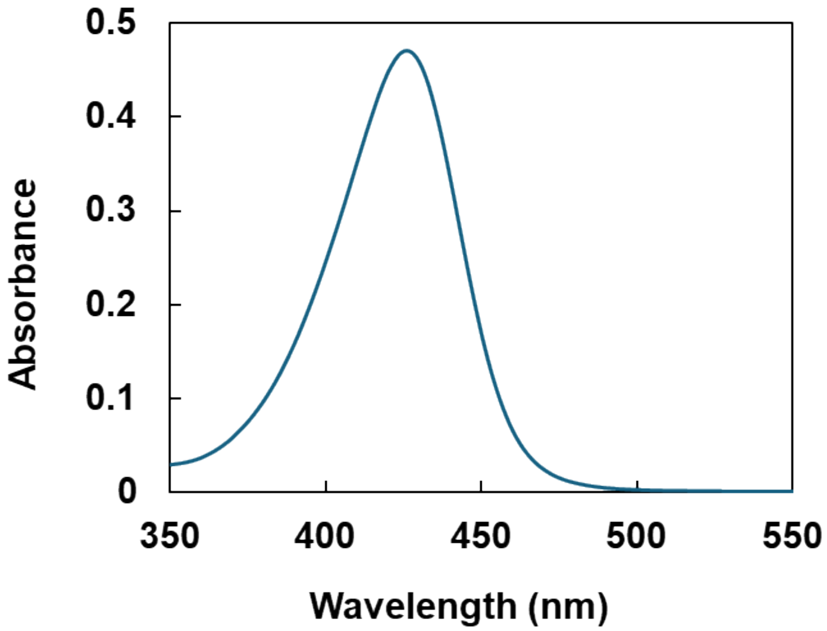

Figure S6. Absorption spectrum of HAPEOP in toluene.
